# Anticancer effects of epigallocatechin-3-gallate nanoemulsion on lung cancer cells through the activation of AMP-activated protein kinase signaling pathway

**DOI:** 10.1038/s41598-020-62136-2

**Published:** 2020-03-20

**Authors:** Bing-Huei Chen, Chia-Hung Hsieh, Su-Yun Tsai, Chian-Yu Wang, Chi-Chung Wang

**Affiliations:** 1 Department of Food Science, Fu Jen Catholic University, New Taipei, 24205 Taiwan; 2 Graduate Institute of Biomedical Sciences, China Medical University, Taichung, 40402 Taiwan; 3 Graduate Institute of Biomedical and Pharmaceutical Science, Fu Jen Catholic University, New Taipei, 24205 Taiwan

**Keywords:** Non-small-cell lung cancer, Non-small-cell lung cancer

## Abstract

Epigallocatechin-3-gallate (EGCG), a green tea–derived polyphenol, exhibits antitumor activities. An EGCG nanoemulsion (nano-EGCG) was prepared to improve the stability and reduce the side effects of EGCG for treatment of human lung cancer cells, and the antitumor effects were studied. The possible molecular mechanism underlying its antitumor effects on cultured human lung cancer cells was also elucidated. The antitumor effects of EGCG and nano-EGCG were determined using methylthiazolyldiphenyl-tetrazolium bromide (MTT), colony formation, migration, and invasion assays. In addition, changes in the AMP-activated protein kinase (AMPK) signaling pathway were investigated using Western blot analyses. AMPK inhibitors were used to determine the roles of the AMPK signaling pathway involved in the molecular mechanism of the nano-EGCG. Our results showed that both EGCG and nano-EGCG inhibited the growth of H1299 lung cancer cells, with half-maximal inhibitory concentrations of 36.03 and 4.71 μM, respectively. Additionally, nano-EGCG effectively suppressed lung cancer cell colony formation, migration, and invasion in a dose-dependent manner. Nano-EGCG may inhibit lung cancer cell invasion through matrix metalloproteinase (MMP)-2- and MMP-9-independent mechanisms. Furthermore, the expression of several key regulatory proteins in the AMPK signaling pathway was modulated by nano-EGCG. Nano-EGCG may inhibit lung cancer cell proliferation, colony formation, migration, and invasion through the activation of AMPK signaling pathways. This novel mechanism of nano-EGCG suggests its application in lung cancer prevention and treatment. Our results provide an experimental foundation for further research on its potential activities and effects *in vivo*.

## Introduction

Lung cancer is established globally to be the leading cause of cancer-related mortality. From 1982 to 2017, in Taiwan, malignant tumors were reported to constitute the principal cause of death. A Taiwan Ministry of Health-issued statistical report revealed that lung cancer ranks first and contributes 23.1% of the standardised mortality rate. In the United States, the estimated number of new lung cancer cases in 2018 was 121,680 in men and 112,350 in women^1^. Among lung cancer cases, non–small-cell lung carcinoma (NSCLC) accounts for 80–85%, with most patients generally presenting with advanced lung cancer. Diagnosing lung cancer in the early stage is difficult, and patients with advanced lung cancer have an only 5% 5-year survival rate^1^. In lung cancer patients, the high potential of invasion as well as that of metastasis to distant organs can explain the aforementioned high mortality rate.

Being a well-conserved energy sensor, the AMP–activated protein kinase (AMPK) has a major function in cellular energy homeostasis maintenance^2^. Tumor cells can rapidly grow and divide; thus, considerable energy is required. AMPK, as demonstrated by previously executed studies, inhibits all cell growth–promoting anabolic pathways^3,4^. In addition, liver kinase B1 (LKB1), representing an established tumour suppressor, functions as an upstream AMPK kinase. Previously executed research has detected LKB1 mutations in inherited cancer disorders and lung cancers, which suggests that AMPK plays a role in tumour suppression^5,6^. AMPK has been considered as a potential therapeutic and prognostic target for lung cancer. For instance, in NSCLC tumors, the expression of AMPK pathway proteins is inversely correlated with recurrence^7^. In addition, in patients with NSCLC, high expression of phosphorylated AMPK was reported to be strongly associated with the lengthening of recurrence-free and overall survival^8^. Evidence also suggests that mutations in LKB1 may lead to unsuppressed cell proliferation because of the inability to activate AMPK in response to the tumor^9^.

Although no drug exists for curing cancer, more than two-thirds of human cancer cases can be prevented by appropriate lifestyle modifications, such as the consumption of dietary-derived agents^10^. Previous studies have implicated bioactive flavonoid compounds in the prevention of human carcinogenesis, possibly through their inhibitory effects on cell proliferation and survival^11,12^. Epigallocatechin-3-gallate (EGCG), which constitutes the principal constituent of green tea, exhibits antiproliferative, anti-inflammatory, antimutagenic, and antioxidative activities^12–15^. EGCG can strongly engender apoptosis and inhibit growth in several types of cancers, including colon, kidney, breast, and brain cancers as well as leukaemia, as demonstrated by *in vivo* and *in vitro* research^10^. However, little information has been reported on the effectiveness of EGCG in lung cancer treatment^16^. Although EGCG can inhibit the growth of small-cell lung cancer cells, it exhibits variable effects on the small number of NSCLC cell lines tested^17,18^.

The efficacy of EGCG *in vivo* is inconsistent with the efficacy *in vitro*; this disparity may be attributed to the weak targeting ability and low bioavailability of EGCG in cancers^19,20^. Recently introduced nanotechnology has been used to increase the bioavailability of chemopreventive agents, including EGCG^21,22^. If a nanoemulsion is prepared to encapsulate EGCG, the bioavailability, stability, and biological activity of EGCG can be considerably enhanced^21^. Moreover, the effective concentration of EGCG can be considerably reduced to minimise its side effects^21^. Therefore, in our study, an EGCG nanoemulsion (nano-EGCG) was prepared, and the antitumour effects exerted on human lung cancer cells by the prepared nano-EGCG were investigated. In addition, the underlying molecular mechanisms of nano-EGCG in lung cancer cells were identified.

## Materials and methods

### Preparation of EGCG nanoemulsion

EGCG standard was purchased from Sigma-Aldrich Co. (St. Louis, MO, USA). The nano-EGCG used in this study was freshly prepared. The details of the preparation procedure were described previously^21^. In brief, a portion of the EGCG standard was poured into a tube, followed by evaporation to dryness under nitrogen, and then 0.05 g of lecithin (0.5%) was added and stirred. Subsequently, 0.5 g of Tween 80 (5%) was added, and the mixture was stirred again; finally, 9.45 g of deionized water (94.5%) was added. After mixing homogeneously, this mixture was shaken in a sonicator for 1.5 hours to obtain a transparent EGCG nanoemulsion with yellow appearance. For the nanoemulsion stability determination, the nanoemulsion was stored at 4 °C for 120 days, during which the particle size and the polydispersity index (PDI) were measured on days 0, 7, 60 and 120^21^. The nanoemulsion size was determined through dynamic light scattering and transmission electron microscopy (TEM) analyses. In addition, the zeta potential and encapsulation efficiency of the EGCG nanoemulsion were calculated using a zeta potential analyzer and a formula described previously^22^. A minor change in particle size, PDI, zeta potential and encapsulation efficiency was shown for the EGCG nanoemulsion over a 120-day storage period^21^.

### Cell culture

Human lung cancer cell lines, namely H1299 (ATCC CRL-5803), A549 (ATCC CCL-185), and primary immortalized bronchial epithelial cell line BEAS2B (ATCC CRL-9609), were maintained in an incubator containing 5% CO_2_ at a temperature of 37 °C and a relative humidity of 100%. The cells were cultured in an RPMI 1640 medium (Life Technologies, MD, USA) containing 10% heat-inactivated fetal bovine serum (FBS; Life Technologies, MD, USA) and 1% penicillin–streptomycin (Life Technologies, MD, USA).

### Cell viability assay

The cells were seeded into 96-well plates at a density of 4,000 cells per well in the culture media. After the cells were cultured with EGCG or nano-EGCG solutions of various concentrations for the indicated durations, cell numbers were measured through thiazolyl blue tetrazolium bromide (also known as methylthiazolyldiphenyl-tetrazolium bromide, [MTT]) assay according to protocol (Sigma-Aldrich Co., St. Louis, MO, USA). The absorbance value at 570 nm was measured using a multiwell scanning spectrophotometer. The MTT assay was used to determine the noncytotoxic concentrations of the nano-EGCG solutions.

### Colony formation assay

For the anchorage-dependent growth assay, 200 cells were resuspended in the RPMI 1640 medium and seeded in six-well plates. The culture media only or nanoemulsion added in media without (controls) or with various concentrations of EGCG or nano-EGCG solutions were changed every 2–3 days. After 7–10 days, the media were removed and the cells were washed and fixed with 4% paraformaldehyde. The fixed cells were stained with 0.05% crystal violet. Colonies >0.8 mm were counted under an inverted microscope. For the anchorage-independent growth assay, the bottom layer contained 0.7% agarose in RPMI 1640, and the top layer contained 0.35% agarose. Cells were seeded at a density of 1,500 cells per well in a six-well plate. The culture media or blank nanoemulsion without (controls) or with various concentrations of EGCG or nano-EGCG solutions were changed every 2–3 days. The plates were incubated at 37 °C with 5% CO_2_ for 4 weeks and subsequently stained with crystal violet. Colonies >0.5 mm were counted under an inverted microscope. The colony formation was assessed in duplicate for three independent experiments.

### Cell migration assay

The cells were seeded into 6-cm culture dishes at a density of 3 × 10^6^ cells per well and cultured for 24 hours in a medium containing 10% FBS. Subsequently, the nearly confluent cell monolayer was carefully scratched using a 10-μL pipette tip. Any cellular debris was removed by washing with phosphate-buffered saline. After being wounded, the cultures were incubated with various concentrations of EGCG or nano-EGCG solutions at 37 °C. At the indicated times (0, 4, 8, and 12 hours) after scraping, the cells were washed twice and immediately photographed. The number of cells migrating into the cell-free zone was counted through a light microscope. In addition, the effects of AMPK inhibitor (BML-275; Enzo Life Sciences, Inc., Farmingdale, NY, USA) on cell migration capabilities were evaluated. All experiments were performed in triplicate.

### Matrigel invasion assay

The invasiveness of tested cells treated with various concentrations of EGCG or nano-EGCG solutions was examined in a Transwell assay using chambers (8-μm pore size; Corning Costar, Cambridge, MA, USA) and Transwell filters coated with Matrigel (BD Biosciences, Franklin Lakes, NJ, USA), as described previously^23^. The number of cells attached to the lower surface of the polycarbonate filters was determined under a light microscope at 400× magnification. All experiments were performed in triplicate.

### Gelatin zymography assay

Cells were seeded at a density of 1 × 10^6^ cells per well in six-well plates for 24 hours. Subsequently, the tested cells treated with various concentrations of EGCG or nano-EGCG was cultured in serum-free media for another 24 hours. To detect matrix metalloproteinase (MMP)-2/9 activities, conditioned media were prepared without boiling or reduction and subjected to sodium dodecyl sulfate–polyacrylamide gel electrophoresis with gels containing 0.1% gelatin. After electrophoresis, the gels were washed with 2.5% Triton X-100 for 30 min and incubated in a developing buffer (50 mM Tris–HCl, pH 8.0, 0.2 M NaCl, 5 mM CaCl_2_, 0.02% Brij35) at 37 °C for 24 hours. Finally, the gels were stained with Coomassie Brilliant Blue R-250.

### Western blot analysis

Western blot analysis was used to examine the expression levels of the affected proteins after nano-EGCG treatments of the tested cell lines. The details of these procedures were described previously^24^. The specific primary antibodies against protein kinase B (Akt), p-Akt, LKB1, p-LKB1, AMPK, p-AMPK, mammalian target of rapamycin (mTOR), p-mTOR, p-P70 (Thr), p-P70 (Ser), and phosphorylated 4E-binding protein 1 (p-4EBP1; Cell Signaling Technology, Danvers, MA, USA) were used for detection, and glyceraldehyde 3-phosphate dehydrogenase (GAPDH) was used as the internal control. After incubation with the primary antibodies, the membranes were washed three times with a solution of Tris-buffered saline and Tween 20. Subsequently, the membranes were incubated with horseradish peroxidase–conjugated secondary antibodies (Santa Cruz, Biotech Inc., CA, USA), and detection was conducted using an enhanced chemiluminescence detection system (ECL, GE Healthcare, NJ, USA).

### Statistical analysis

All experiments were performed in triplicate and analyzed through analysis of variance (Excel, Microsoft) to determine significant differences. Where appropriate, the results are expressed as the mean ± standard deviation (SD). All statistical tests were two-sided, and *P* values <0.05 were considered statistically significant.

## Results

### Effects of Nano-EGCG on lung cancer cell proliferation activity

**Figure 1:**
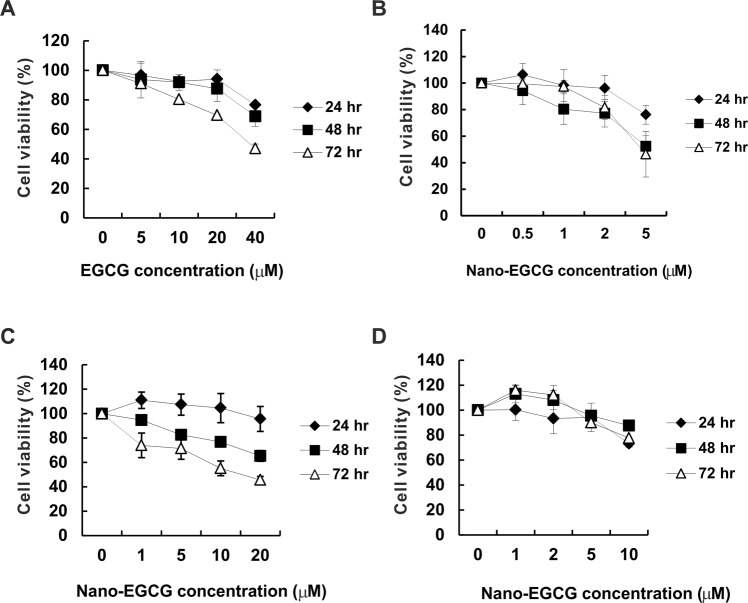
Effects of EGCG and nano-EGCG on the viability of H1299, A549, and BEAS2B cells. H1299 cells were treated with different concentrations of EGCG (**A**) or nano-EGCG (**B**) for the indicated time periods, and the subsequent cell viability was measured through MTT assay. The cell viability of A549 (**C**) and BEAS2B (**D**) cells in response to nano-EGCG was also assessed. The number of viable cells after treatment is expressed as a percentage of the control group (culture media or nanoemulsion without EGCG). These results are representative of two independent experiments performed at least in triplicate. *P < 0.05, significantly different from the control group at the indicated time. Data are expressed as mean ± SD.

EGCG was reported to exhibit an antiproliferative effect on lung cancer cells^16^. To study the effects of EGCG and nano-EGCG on human lung cancer cells, we first determined whether EGCG or nano-EGCG at the indicated concentrations of treatments for 24, 48, and 72 hours could influence the viabilities of H1299, A549, and BEAS2B cells. After treatment, we conducted an MTT assay to determine the cell viabilities (Fig. 1). A dose-dependent decrease was demonstrated in the H1299 cell viability after treatment with EGCG or nano-EGCG for 72 hours (Fig. 1A,B). We discovered that EGCG could suppress H1299 cell proliferation at doses higher than 20 μM. However, only 5 μM doses of nano-EGCG could significantly inhibit H1299 cell viability. Comparatively, the half-maximal inhibitory concentration (IC_50_) of EGCG and nano-EGCG for H1299 lung cancer cells was 36.03 μM and 4.71 μM, respectively. Nano-EGCG exhibited more efficient inhibition than did EGCG of the growth of H1299 cells. In addition, the effects of nano-EGCG on the growth of another lung cancer cell, A549, were determined. As indicated in Fig. 1C, A549 cell viability followed a dose-dependent decline after nano-EGCG treatment for 48 and 72 hours. The IC_50_ of nano-EGCG for A549 cells was 16.05 μM. To further clarify whether nano-EGCG could influence the growth of lung epithelial cells, the viability of BEAS2B cells was detected. With a nano-EGCG dose of <5 μM, no significant decrease was exhibited in BEAS2B cell viability after the indicated time periods (Fig. 1D). When the nano-EGCG doses were raised to 5 μM and 10 μM, the viability of BEAS2B cells at 72 hours decreased to 90.17% and 77.72%, respectively. Thus, nano-EGCG exhibited greater antiproliferative activity in H1299 and A549 human lung cancer cells than in BEAS2B cells.

### Effects of EGCG and nano-EGCG on lung cancer cell colony formation activity

**Figure 2:**
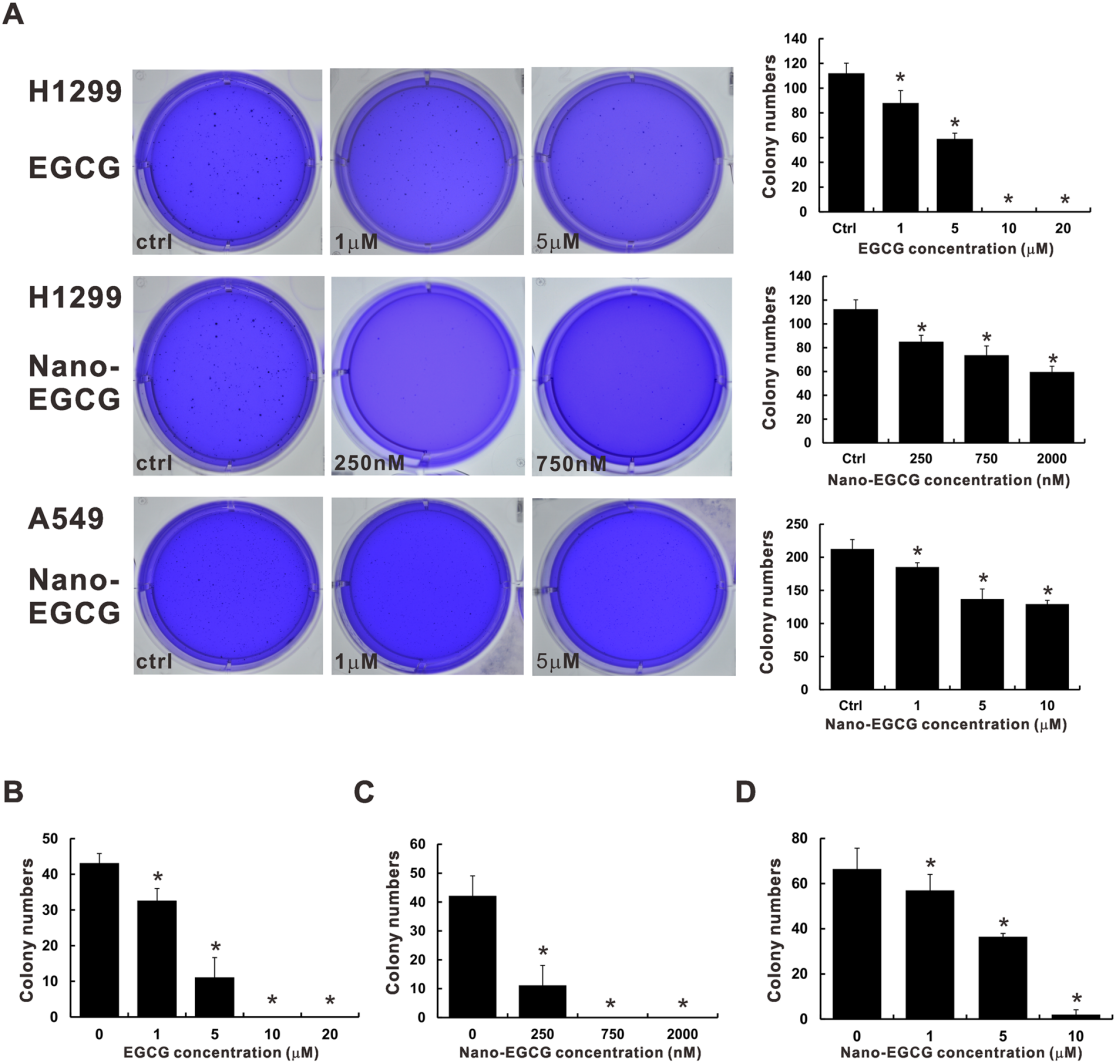
Effects of EGCG and nano-EGCG on the colony formation activity in lung cancer cells. (**A**) Representative images of the anchorage-independent colony formation assay in H1299 and A549 cells. The graphs are the summarized data of the colony formation assays. Colonies >0.5 mm in diameter were counted and displayed in comparison with the control group. Anchorage-dependent colony formation assays of H1299 cells treated with EGCG (**B**) and nano-EGCG (**C**). (**D**) The effects of nano-EGCG on A549 cells anchorage-dependent colony formation abilities were also studied. Colonies >0.8 mm in diameter were counted and displayed in comparison with the control group. Values are reported as means ± SD (*n* ≥ 3). *P < 0.05 compared with the control group.

Subsequently, the effect of nano-EGCG on the colony formation ability of lung cancer cells was determined using anchorage-independent and -dependent colony formation assays. As displayed in Fig. 2A, both H1299 and A549 cells treated with nano-EGCG formed fewer anchorage-independent colonies than did the control cells. Quantitative data revealed that nano-EGCG inhibited the anchorage-independent colony formation activity of the H1299 and A549 cells in a concentration-dependent manner. Treatment with nano-EGCG at a low dose (1 μM) significantly reduced the number of colonies formed in the A549 cells to 86% relative to the control (*P* < 0.05), whereas treatment with higher concentrations of nano-EGCG (20 μM) significantly inhibited colony formation in the A549 cells. However, unlike with EGCG, the colony formation activity was significantly inhibited at doses as low as 250 nM nano-EGCG for H1299 cells (*P* < 0.05). With EGCG doses >5 μM, the colony formation activity of H1299 cells was completely suppressed (Fig. 2A,B). The results of the anchorage-dependent colony formation assay revealed that nano-EGCG doses of 250 nM and 1 μM could significantly inhibit the colony formation abilities of H1299 and A549 cells, respectively (Fig. 2C,D; *P* < 0.05).

### Effects of nano-EGCG on lung cancer cell migration and invasive activity

**Figure 3:**
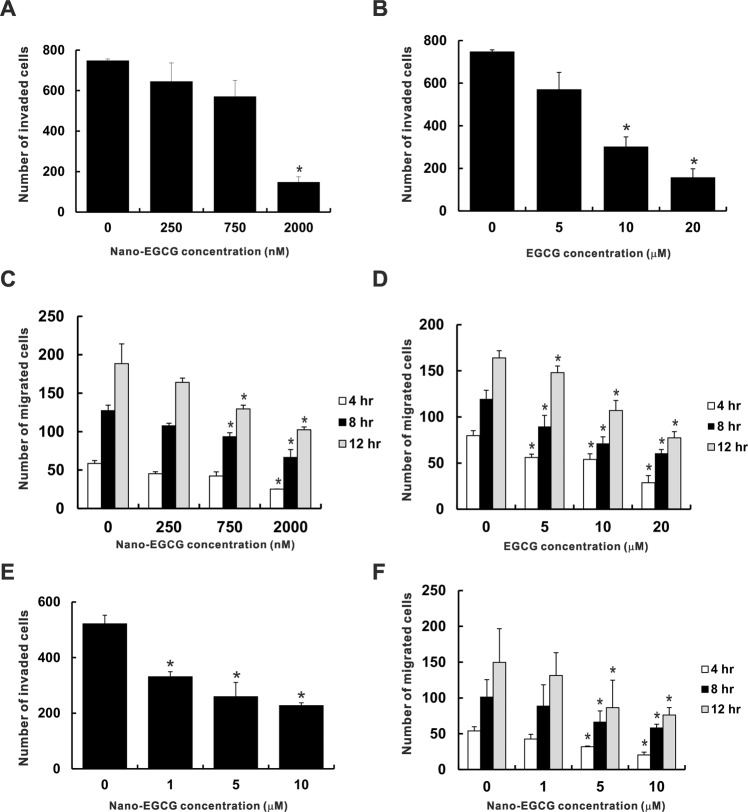
Effects of EGCG and nano-EGCG on the cell invasion and migration of H1299 and A549 cells. The effects of different concentrations of nano-EGCG (**A**) and EGCG (**B**) on the invasive capabilities of H1299 cells were determined. The cell migration ability of H1299 cells treated with nano-EGCG (**C**) or EGCG (**D**) was assessed through scratch wound-healing assays. The number of cells that migrated into the cell-free zone was evaluated at 4, 8, and 12 hours after wounding. The effects of nano-EGCG on the cell invasion (**E**) and migration (**F**) abilities of A549 cells were also determined. The data are representative of three independent experiments and are indicated as the mean ± SD. *P < 0.05 compared with the control group.

Because tumor metastasis is the main impediment to treating lung cancer, we considered whether nano-EGCG could inhibit the migration and invasion capabilities of lung cancer cells. As indicated in Fig. 1B,C, nano-EGCG did not greatly affect the cell viabilities of H1299 and A549 after 24 hours at doses lower than 5 μM and 20 μM, respectively. Thus, the effect of nano-EGCG on cell invasion of H1299 was determined through Transwell invasion assay to be <5 μM. As illustrated in Fig. 3A, nano-EGCG reduced the invasive ability of H1299 cells in a dose-dependent manner. Treatment with nano-EGCG at 2 μM significantly reduced (*P* < 0.05) the invasion ability of H1299 cells to 19.8% relative to the control. Similar results were discovered in the EGCG groups (Fig. 3B). Higher doses of EGCG (10 and 20 μM) could significantly inhibit the invasion capability of H1299 cells. In addition, the effect of nano-EGCG on lung cancer cell migration was evaluated using a wound-healing assay. Nano-EGCG could inhibit the migration of H1299 cells both in concentration- and time-dependent manners (Fig. 3C). Similar results were also discovered in the EGCG groups (Fig. 3D). However, the effective inhibitory concentration of nano-EGCG for H1299 cell migration ability was lower than that of EGCG. Furthermore, nano-EGCG inhibited the migration and invasion capabilities of A549 cells in a dose-dependent manner, as illustrated in Fig. 3E,F (*P* < 0.05). These results revealed that nano-EGCG could suppress lung cancer cell migration and invasion at low doses.

### Effects of nano-EGCG on MMP-2 and MMP-9 activities in lung cancer cells

**Figure 4:**
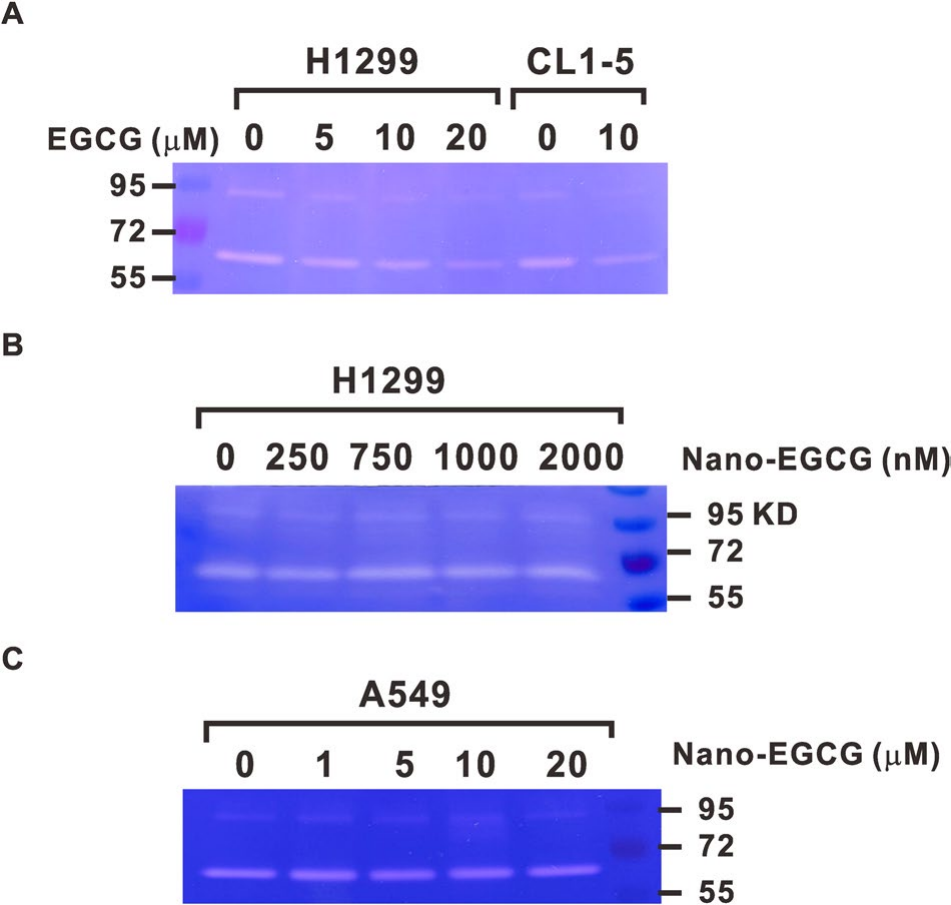
Effects of EGCG and nano-EGCG on the activation of MMP-2 and MMP-9 in lung cancer cells. (**A**) H1299 and CL1-5 cells were treated with EGCG in serum-free medium for 24 hours, and the activity of MMPs was analyzed through a gelatin zymography assay. (**B**) H1299 and (**C**) A549 cells were treated with different concentrations of nano-EGCG before the subsequent zymography assay.

A previous study demonstrated that EGCG could repress the activities of MMP-2 and MMP-9 in highly invasive CL1-5 lung adenocarcinoma cells^25^. Thus, to determine whether nano-EGCG has any effects on MMP-2 and MMP-9 in lung cancer cells, we first collected conditioned media of H1299 and CL1-5 cells treated for 24 hours with various concentrations of EGCG or nano-EGCG and analyzed the media through gelatin zymography assay. In accordance with previous results^25^, the gelatin-degrading activity significantly decreased in the presence of EGCG at 10 μM in CL1-5 cells. In addition, EGCG could reduce the activities of MMP-2 and MMP-9 in H1299 cells in a dose-dependent manner (Fig. 4A). However, nano-EGCG had no significant effects on the activities of MMP-2 and MMP-9 in either H1299 or A549 cells (Fig. 4B,C). These results suggested that nano-EGCG could suppress the invasion activity of lung cancer through MMP-2- and MMP-9-independent mechanisms; this is different from how EGCG acted on lung adenocarcinoma cells.

### Effects of nano-EGCG on AMPK signaling in lung cancer cells

**Figure 5:**
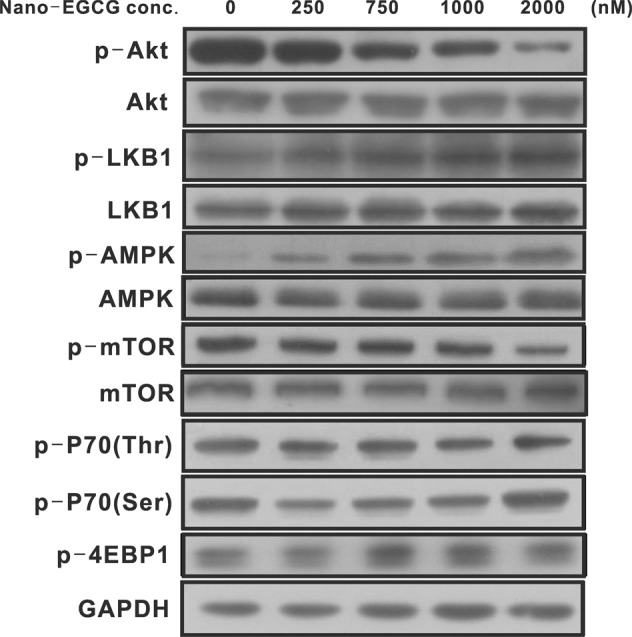
Immunoblot analysis revealed the effects of nano-EGCG treatment on AMPK-related pathway gene expression levels. The expression levels of Akt, p-Akt, LKB-1, p-LKB-1, AMPK, p-AMPK, mTOR, p-mTOR, p-P70, and p-4EBP1 proteins in the H1299 cells that were treated with different concentrations of nano-EGCG were determined through western blot analysis. The nanoemulsion without EGCG was used as a control group. GAPDH was used as an internal control for protein loading and transfer. The original blots/gels are presented in Supplementary Information.

As previously mentioned, nano-EGCG could significantly inhibit the proliferation, colony formation, migration, and invasion activities of the H1299 and A549 cells. However, the signal transduction mechanisms responsible for the inhibitory effects of nano-EGCG remained unknown. The activation of AMPK has been reported to inhibit tumor progression in several types of cancers; therefore, we further assessed the effects of nano-EGCG on the signal transductions of AMPK in lung cancer cells through western blot analysis. The H1299 cells were treated with various concentrations of nano-EGCG for 48 hours. Subsequently, the total protein lysates of each sample were collected and subjected to western blotting with various total protein or phosphoprotein antibodies. As illustrated in Fig. 5, nano-EGCG treatment significantly reduced the phosphorylation of Akt in H1299 cells and significantly increased the LKB1 and AMPK phosphorylation levels. Furthermore, the phosphorylation levels of downstream target proteins of AMPK, such as mTOR, P70, and 4EBP1, were also regulated by nano-EGCG treatment. In summary, the inhibitory effects of nano-EGCG on the proliferation and invasion of the lung cancer cells might result from activation of the AMPK signaling pathway.

### Effects of nano-EGCG and AMPK inhibitor on migration capability of lung cancer

**Figure 6:**
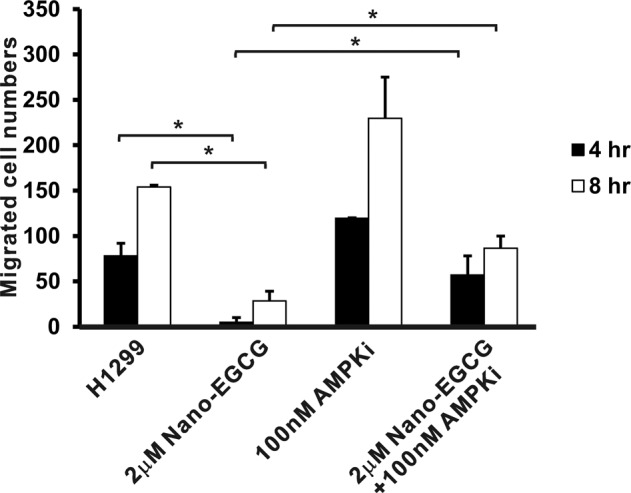
Effects of Nano-EGCG and AMPK inhibitors on H1299 cell migration. H1299 cells were seeded in the chamber of a 6-cm dish and incubated for 1 day before the addition of Nano-EGCG (2 μM) and AMPK inhibitors (BML-275, 100 nM). The wound healing assay was performed and photographs were taken at 0, 4, and 8 hours after treatment. The total numbers of migrated cells in the denuded zone were counted. These results are representative of two independent experiments performed in triplicate. *P < 0.05 significantly different from the indicated control group at the indicated time. Data are expressed as mean ± SD.

**Figure 7:**
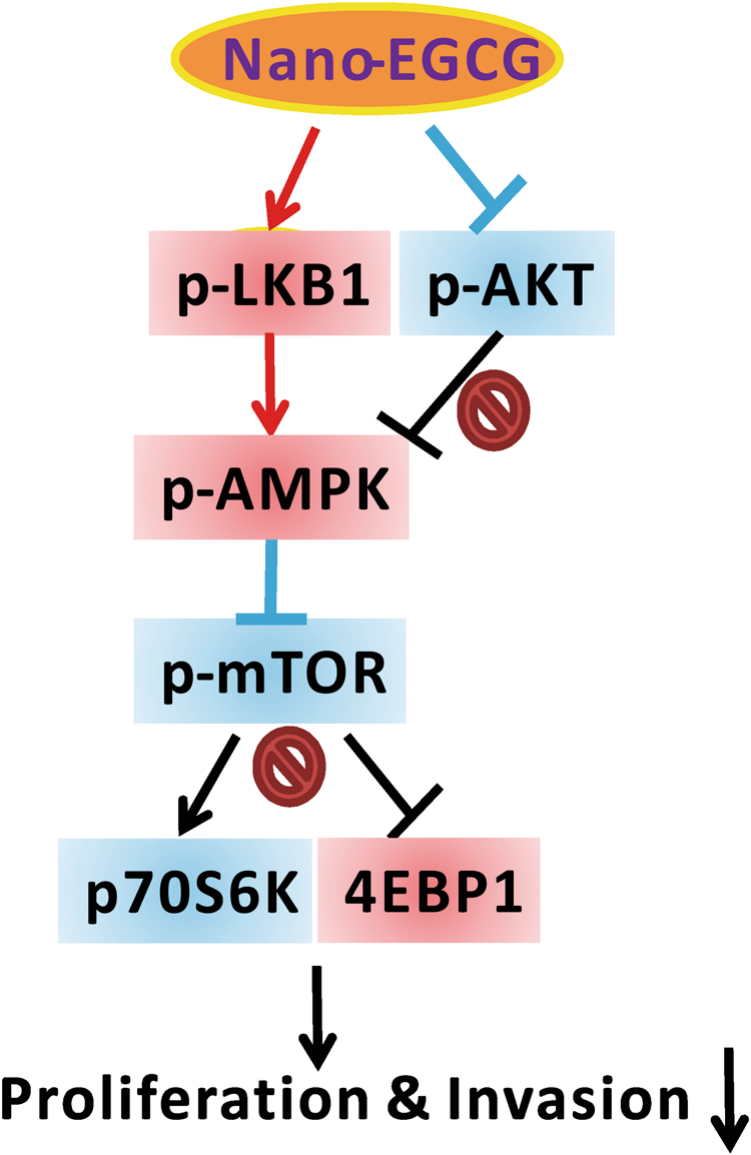
A hypothetical model of how nano-EGCG causes AMPK activation and related signaling events while exhibiting anticancer effects. Nano-EGCG may activate AMPK expression through Akt inactivation and LKB-1 activation to suppress lung cancer cell proliferation, migration, and invasion. Red color indicates gene activation or upregulation and blue color means gene inactivation or downregulation after nano-EGCG treatment.

To further confirm that AMPK plays a major role in reducing the migration ability regulated by nano-EGCG in H1299 cells, the inhibitor of AMPK, BML-275, was used. H1299 cells were pretreated with 2 μM nano-EGCG, 100 nM AMPK inhibitor, or a combination and then subjected to wound-healing migration assays. As indicated in Fig. 6, 2 μM nano-EGCG could significantly suppress the migration activity of H1299 cells compared with control groups (*P* < 0.05). However, the AMPK inhibitor treatment could slightly increase the numbers of migrated H1299 cells. Moreover, the treatment of H1299 cells with combined nano-EGCG and AMPK inhibitor could significantly reverse the inhibitory effects of nano-EGCG on cell migration capabilities at the indicated time (*P* < 0.05). These results indicated that the AMPK signaling pathway might be involved in nano-EGCG regulated antitumor activities in lung cancer cells (Fig. 7).

## Discussion

Phytochemicals present in certain fruits, vegetables, and tea have attracted scientific attention as potential agents for cancer treatment and prevention. Among various tea products, green tea has received considerable attention; numerous studies have suggested that EGCG, the most abundant constituent of total catechins in green tea, has anticarcinogenic activities^26^. However, the effects of EGCG on human lung cancer are largely unknown. In the present study, to enhance the stability of EGCG and its ability to target human lung cancer cells, an EGCG nanoemulsion was prepared, and the antitumor effects of nano-EGCG on lung cancer cells were determined.

Our data demonstrated for the first time that nano-EGCG can play a crucial role in increasing the AMPK phosphorylation expression and suppressing the proliferation, colony formation, migration, and invasion capabilities of lung cancer cells by activating the AMPK signaling pathway. Notably, nano-EGCG could suppress the invasion activity of lung cancer through MMP-2- and MMP-9-independent mechanisms; this differs from how EGCG was reported to exert effects on lung adenocarcinoma cells^25^. Our results were consistent with previous studies on breast cancer cells^27^, according to which the antitumor effectiveness of nano-EGCG is superior to that of EGCG on lung cancer cells. Furthermore, the blockage of activated AMPK in lung cancer cells could significantly reverse the inhibitory effects of nano-EGCG on cell migration activities. Taken together, our results indicate that the activated AMPK signaling pathway induced by nano-EGCG treatment is involved in antitumor activities in human lung cancer cells.

Catechins have been reported to have several biological benefits. EGCG is the major constituent of catechins present in green tea. Previous studies have suggested that EGCG exhibits a variety of activities, such as antiobesity, antidiabetes, anti-inflammatory, and antitumor activities^26^. EGCG could inhibit carcinogenic activity, proliferation, angiogenesis, migration, invasion, and tumorigenesis and induce cell death. These antitumor effects are associated with the modulation of several signaling molecules, including reactive oxygen species, NF-κB, Akt, vascular endothelial growth factor, peroxisome proliferator-activated receptor, Bcl-2, and mitogen-activated protein kinases, as well as epigenetic modification^28^. Although numerous studies have demonstrated that EGCG exhibits health-promoting effects at a certain dose, toxicity of EGCG has also been reported. An overdose of green tea or EGCG on rat models caused adverse complications such as hepatotoxicity through liver enzyme alterations^29^. In addition, previous studies have demonstrated that EGCG is lower in plasma than the actual ingested quantity, thus indicating low bioavailability^30^. Therefore, nanotechnology was recently introduced to improve bioavailability and reduce the size of doses of EGCG.

For example, chitosan nanoparticles encapsulating EGCG have been reported to possess numerous significant biological and chemical properties such as biocompatibility, hemostasis, low toxicity, biodegrability, and anticarcinogenesis^31^. Our data revealed that nano-EGCG could achieve similar anticancer to EGCG effects at a lower concentration. The IC_50_ of nano-EGCG in inhibiting H1299 cells was much lower than the EGCG standard (4.71 μM vs. 36.03 μM). However, nano-EGCG could not drastically reduce the viability of BEAS2B bronchial epithelial cells at this low dose. In addition, low concentrations of nano-EGCG could significantly inhibit the colony formation, migration, and invasion abilities of lung cancer cells. A previous study suggested that EGCG could inhibit the invasion of CL1-5 lung cancer cells by suppressing MMP-2 expression through c-Jun N-terminal kinase signaling^25^. However, our results demonstrated that nano-EGCG might inhibit lung cancer cell invasion through an MMP-2- and MMP-9-independent pathway. Although EGCG could suppress the MMP-2 and MMP-9 activities in H1299 cells and CL1-5 cells, the inhibitory effects on MMP activity were lost on the H1299 and A549 cells treated with nano-EGCG (Fig. 4). The different characteristics of these two biomaterials require further investigations.

EGCG has been reported to play a role in the management of cancer through the modulation of different cell signaling pathways. For example, previous studies have demonstrated that EGCG could induce the expression of p53 and Bax in prostate cancer and breast cancer cells^32,33^. Other findings have suggested that EGCG could inhibit the activation of Akt in bladder cancer and breast cancer cells^34,35^. Furthermore, the activation and expression of c-Met; MAPK; and transcription factors activator protein 1, NF-kB, and Stat3 were reported to be modulated by EGCG^28^. AMPK is known to be involved in cellular homeostasis, and AMPK plays a crucial role in cell survival and apoptosis. EGCG could suppress colon cancer proliferation by modulating the AMPK activation^36^. The loss of function of LKB1, an upstream kinase of AMPK, in lung adenocarcinoma is 30–50%^37^. Because of the discovery of the antineoplastic effects of AMPK, the number of patents for potential AMPK activators has grown rapidly^38,39^. However, in some cases, AMPK activation may promote tumor survival through mTORC2/PI3K-Akt signaling pathway activation^40^.

The data in the present study revealed that nano-EGCG not only induces LKB1 and AMPK activation but also suppresses the activation of Akt. However, the study also has several limitations. First, the effect of nano-EGCG on Ca^2+^/CaM-dependent protein kinase kinase β, another upstream activator of AMPK, requires investigation in further studies. Moreover, although *in vitro* experiments were performed in the current study, the effects of nano-EGCG *in vivo*, such as tumorigenesis and metastasis, should be determined. Finally, whether nano-EGCG could improve the targeting ability and bioavailability *in vivo* requires further investigation.

## Conclusions

Our results demonstrated for the first time that significant inhibition of proliferation, colony formation, migration, and invasion of human lung cancer cells modulated through the activation of the AMPK signaling pathway by low doses of nano-EGCG. Therefore, nano-EGCG could be developed as a potential antitumor candidate targeting AMPK for the prevention and treatment of lung cancer. In addition, the potential clinical use of this agent warrants further evaluation and investigation. The use of low doses of nano-EGCG, which provides low toxicity and high efficacy, in adjuvant therapies with other agents may be more promising for lung cancer therapy.

## Supplementary information

Supplementary information.

## Data Availability

The obtained results of the research are available on reasonable request.
