# Supplementary material for: Anticancer effects of epigallocatechin-3-gallate nanoemulsion on lung cancer cells through the activation of AMP-activated protein kinase signaling pathway
Source: Sci Rep. 2020 Mar 20;10:5163. doi: 10.1038/s41598-020-62136-2 (PMC7083948; doi:10.1038/s41598-020-62136-2)
Supplement: Supplementary file 1 — Supplementary information. [file 41598_2020_62136_MOESM1_ESM.pdf]

SUPPLEMENTARY INFORMATION

# Anticancer effects of epigallocatechin-3-gallate nanoemulsion on lung cancer cells through the activation of AMP-activated protein kinase signaling pathway

Bing-Huei Chen[1§], Chia-Hung Hsieh[2§], Su-Yun Tsai[3], Chian-Yu Wang[3], Chi-Chung Wang[3*]

[1]Department of Food Science, Fu Jen Catholic University, New Taipei, 24205, Taiwan.
[2]Graduate Institute of Biomedical Sciences, China Medical University, Taichung, 40402, Taiwan.
[3]Graduate Institute of Biomedical and Pharmaceutical Science, Fu Jen Catholic University, New Taipei, 24205, Taiwan.

§ These authors contributed equally to this study.

*** Corresponding author:** Chi-Chung Wang, Ph.D., Graduate Institute of Biomedical and Pharmaceutical Science, Fu Jen Catholic University, No. 510, Zhongzheng Rd., Xinzhuang Dist., New Taipei City, 24205, Taiwan (R.O.C.) Phone: 886-2-29052039, Fax: 886-2-29053415, Email: 075006@mail.fju.edu.tw.

**Original Western Blots**

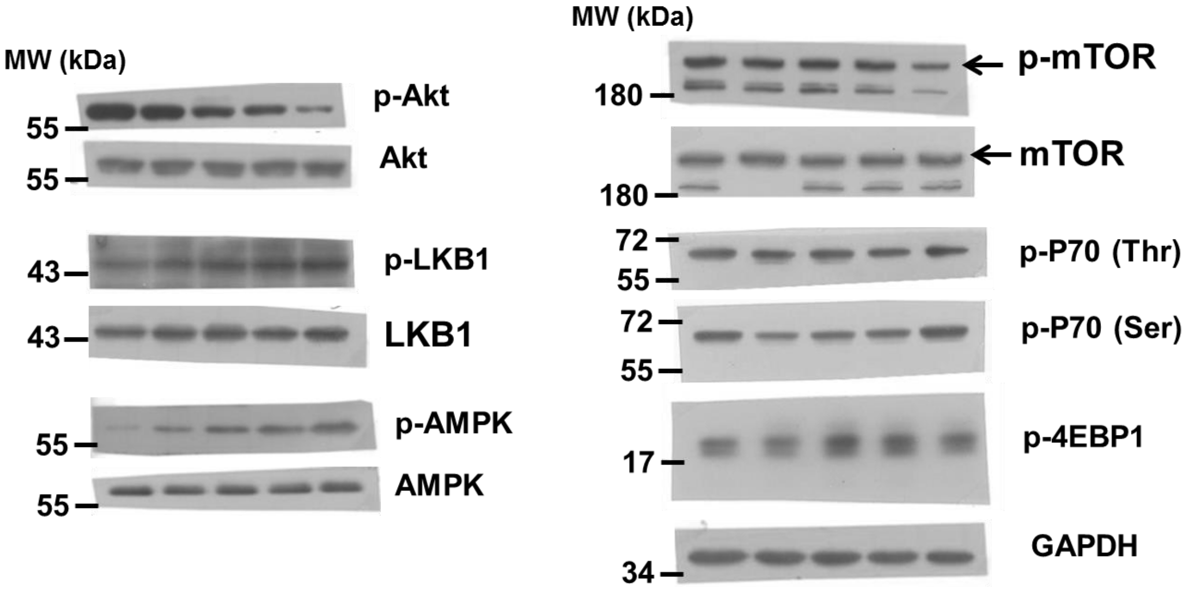


Fig. 5 Immunoblot analysis revealed the effects of nano-EGCG treatment on AMPK-related pathway gene expression levels. In our lab, different parts of the same blot were used to be cropped for different antibodies that hybridized to the indicated genes at the same time.
